# A scoping review of intensive longitudinal methods in informal caregivers of people with dementia

**DOI:** 10.1186/s12877-023-04123-6

**Published:** 2023-07-25

**Authors:** P Gérain, E Wawrziczny, P Antoine

**Affiliations:** 1 Department of Psychology, Faculty of Educational and Psychological Sciences, Vrije Universiteit Brussel, Pleinlaan 2, 1050 Brussels, Belgium; 2 SCALAB – UMR 9193, University of Lille, Lille, France

**Keywords:** Informal caregivers, Family carers, Experience sampling, Ecological momentary assessment, Burden, Intensive longitudinal methods

## Abstract

**Background:**

The daily life of informal caregivers assisting individuals with dementia widely varies throughout the day and week. As an answer, an increasing number of researchers have used intensive longitudinal methods (ILMs) such as diary studies, experience sampling methods, or ecological momentary assessment.

**Objectives and Methods:**

The present scoping review aims at synthesizing the use of ILMs in informal dementia caregivers to clarify what is currently done and how, as well as what remains unaddressed.

**Results:**

The screening process identified 48 studies from 22 different datasets. Synthesis of these studies showed the diversity of devices and uses of ILMs in informal care, including the exploration of associations between variables or accompanying an intervention. ILMs showed the important variability of caregiving phenomena, as well as the important association of momentary stress and well-being. Gaps were nevertheless identified, such as transparency in the construction of the tool or the absence of focus on emotions and dyads.

**Conclusions:**

For now, this field of research remains in its infancy and does not seem to have reached its full potential as it has in other fields. Nevertheless, it appears that ILMs are promising tools for informal dementia caregivers as they contribute to understanding the complexity of their daily life, with changing resources and challenges. Future directions include focusing more on (emotion) regulation, temporal lags, and the use of ILMs in interventional designs.

**Trial registration:**

The present review was registered on OSF (osf.io/b2qr4).

**Supplementary Information:**

The online version contains supplementary material available at 10.1186/s12877-023-04123-6.

## Background

A scoping review of intensive longitudinal methods in informal caregivers of people with dementia Informal caregiving refers to providing care to a relative who faces a loss of autonomy due to a disease, a disability, or any health-related condition [1]. An important proportion of informal caregivers assist a person with a form of dementia, and the number of dementia caregivers required to assist people with dementia is expected to significantly rise in the coming years [2]. Dementia care represents a particular challenge due to the complex and evolutive symptomology of dementia. It often starts with small daily challenges (e.g., memory losses) but ends up with more important difficulties affecting daily life (e.g., confusion and wanderings) [3]. In the long run, the caregiver must deal with the evolution of the disease, which requires accepting the inevitable fate of the care-recipient; in daily life, the caregiver must deal with the daily fluctuations of dementia symptomatology, requiring a constant day-to-day adaptation from the caregiver to the care needs of the care-recipient [4*].

For decades, quantitative research in the field has mostly relied on different designs using self-report retrospective questionnaires to understand what affects the informal caregiver’s well-being. Such questionnaires give a subjective observation of symptoms, processes, or behaviors for an individual, over a certain period while the person must recall and then rate how much they experienced it [5]. As such, retrospective questionnaires reflect the perceived experience of the person that has been reconstructed based on their perceptions [6] and consequently suffer several biases, including a retrospective reconstruction bias [7]. Therefore, we face the issue that the use of such methods gives information that may fail to reflect the daily fluctuations of providing care to an individual with dementia.

One of the ways to answer that issue is to focus on the moment by using intensive longitudinal methods (ILMs, 5). ILMs cover a range of methods under different terminologies (e.g., ecological momentary assessment, experience sampling, daily diary). All have in common to use of multiple within-subject subjective assessments in a relatively short time frame (e.g., one or more measurements a day for several consecutive days) [5, 7]. The goal of these methods is to get closer to the lived experience of the individuals, which allows, for example, to explore the dynamics of mood and processes, their fluctuations over time, and if they occurred in certain contexts [7].

ILMs appear particularly relevant to informal care as they allow the investigation of the daily variability of what informal care is. The day-to-day (or even hour-to-hour) life of an informal caregiver could importantly vary. As summarized by Bosch et al. [8], the care load varies according to the changing needs of the care-recipient that fluctuates, and their positive or negative feelings toward informal care widely differ based on time and context. Day-to-day informal care is so diverse that ILMs appear to be a necessary means in that context, to get closer to the daily experience of dementia caregivers. However, as promising as these tools appear, there is currently no clear picture of the use of such methods in informal care. Consequently, there is no synthesis as to what information these methods currently provide, but also no overview of the tools used and why researchers rely on these tools.

### Objectives

To answer this, a scoping review [9] of the existing literature on the use of ILMs in the context of informal dementia care appears necessary. The objective of the present review is to have a synthesis of (a) the purpose of using such methods, (b) how they are implemented, and (c) the results they showed.

## Methods

The present review follows the extension for scoping reviews of the Preferred Reporting Items for Systematic Reviews and Meta-Analyses (PRISMA-ScR) guidelines [10]. A checklist is displayed in Supplementary Materials 1. The protocol of this review was registered on the OpenSourceFramework (reference: osf.io/b2qr4).

### Eligibility criteria

The inclusion criteria were (a) to use any form of intensive longitudinal design (i.e., using multiple self-report measurements in a short timeframe), (b) in the context of providing informal care to a person with dementia or a related disease, and (c) be written in English. Studies were included regardless of their design, publication year, or publication status. They were excluded if they only used an intensive longitudinal design for gathering descriptive data (e.g., sleep hours, activities), without consideration of indicators of behaviors, well-being, or psychological states and processes. This criterion was set to focus on the understanding of processes and causes of fluctuations in daily life.

### Information source and search

The studies were retrieved from five different online databases: PsycInfo, PsycArticle, Pubmed, WebOfScience, and Scopus. These databases cover mostly published manuscripts, but some (e.g., PsycInfo) include a large set of works from the grey literature. Considering that the objective was to make an inventory of the existing publications, no approach was taken to explore the grey literature. Reference lists of included articles were screened to find additional studies. This extraction of online databases was performed in 2021 and updated in March 2022. Keywords used to retrieve studies are presented in Supplementary Materials 2.

### Selection of sources of evidence

**Fig. 1:**
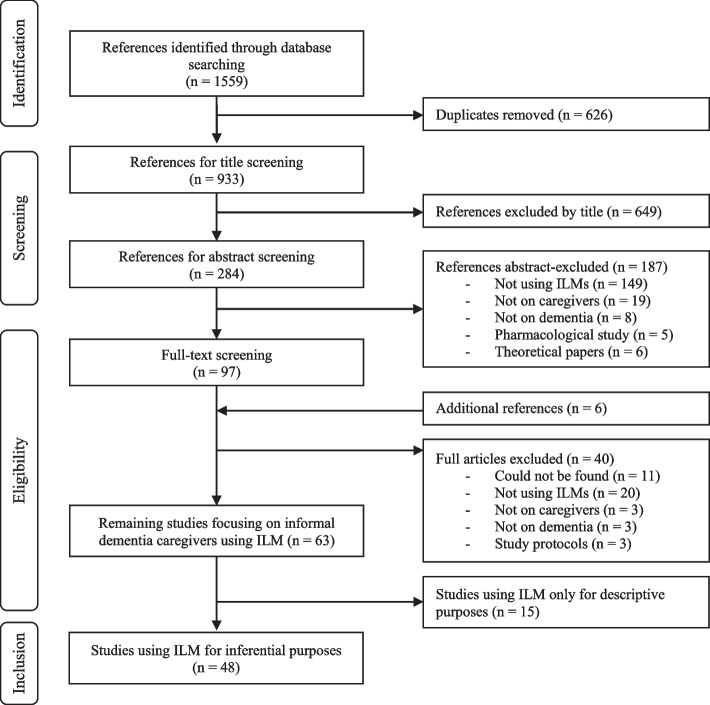
PRISMA flowchart of the inclusion process of the studies

Once the references were extracted from the online databases, they were imported into EndNote X9. First, duplicates were deleted using a de-duplication protocol [11]. Then, studies were first screened based on the title, then on the abstract, then on the full text by the first author with the support of the last author (see Fig. 1). using the inclusion criteria, in the following order: (a) the study is written in English, (b) focuses on dementia informal caregivers, (c) provides empirical evidence, and (d) relies on a form of ILMs (as defined in eligibility criteria).

### Data charting process and data items

The information on the studies was extracted by the main author of the study using an Excel spreadsheet and then reviewed with the other authors. The form was based on a form used for a previous systematic review performed in informal care research on a different topic [12], augmented by items specific to ILMs [13]. As the objective of the study was to have an overview of the literature, the independence or blinding of the data extraction did not appear necessary.

Various information about each study was collected: date of the study, use of the intensive measurement, the purpose of the study, number and kind of participants, the tool used for ILMs, questions asked in the ILMs, baseline questions, analyses made, duration of the ILMs, number of assessments per day, main results, main difficulties encountered (if any).

### Synthesis of results

Following the objective of giving an overview of the use of ILMs in informal care contexts, the synthesis consisted of how ILMs were used, the variables investigated, the tools used, the main results found, as well as the limits and challenges encountered. When the results of a study were reported in several publications, the publication with the most complete data was identified as the primary reference and the other publications were considered as associated references (see Table 1).

**Table 1:** Descriptive summary of the included studies and datasets

Main reference	Country	Number of caregivers	Age	Women	Sampling	Condition of the care-recipient	Other studies
Bartels et al., 2020 [14*]	Netherlands	72	72.1 (8.4)	67%	Memory clinics and institutions	Dementia	[15*–19*]
Fauth et al., 2006 [20*]	USA	85	66.3 (11.6)	75	Respite programs and convenient	Dementia	/
Fonareva et al., 2012 [21*]	USA	18	66.4 (7.8)	89%	Convenient	Dementia	/
Goodridge et al., 2021 [22*]	Canada	53	76.3 (12.9)	91%	Institutions and social media	Dementia	/
Jain et al., 2014 [23*]	USA	10	64.0 (7.0)	100%	Convenient	Dementia	/
Jayalath et al., 2016 [24*]	United Kingdom	78	n.m	n.m	Clinics	Alzheimer's Disease	/
Koerner & Shirai, 2012 [25*]	USA	67	52.9 (9.4)	88%	Clinics and convenient	Cognitive impairment and other conditions	[26*–28*]
Konnert et al., 2017 [29*]	Canada	9	59.7 (9.5)	100%	Clinics and convenient	Residents of a nursing home, 2/3 with dementia	/
Liu et al., 2021 [30*]	USA	165	62.0 (10.7)	88%	Adult day services	Dementia	[31*–43*]
MaloneBeach et al., 1995 [44*]	USA	43	56.74	100%	Clinics and convenient	Dementia	/
Mather et al., 2022 [45*]	USA	40	66.4 (11.8)	83%	n.m	Alzheimer's Disease	[46*]
McCrae et al., 2016 [47*]	USA	55	62.8 (12.2)	78%	From larger parent study	Dementia	/
Monin et al., 2017 [48*]	USA	73	71.5 (10.6)	64%	Clinics and convenient	74% dementia and 26% other conditions	[49*]
Pickering et al., 2020 [50*]	USA	50	53.0 (11.0)	93%	Social and news media	Dementia	/
Pickering et al., 2022 [51*]	USA	64	59.7 (13.4)	84%	Social and news media	Dementia	[50*]
Pihet et al., 2017 [4*]	Switzerland	26	68.0 (median)	77%	Clinics and convenient	Dementia	/
Potts et al., 2020 [52*]	United Kingdom	28	67.0 (13.0)	79%	n.m	Dementia	/
Rullier et al., 2014 [53*]	France	15	n.m	n.m	Cohort of farmers	Retired farmers with and without cognitive impairment	/
Ryuno et al., 2021 [54*]	Japan	25	66.3 (10.8)	72%	Adult day services and nursing homes	Dementia	/
Savla et al., 2013 [55*]	USA	30	72.9 (6.8)	90%	Memory clinics and institutions	Mild cognitive impairment	[56*]
van Knippenberg, de Vugt, Ponds, Verhey, et al., 2018 [16*]	Netherlands	30	69.9 (5.8)	60%	Memory clinics and institutions	Dementia	[17*]
Zawadzki et al., 2021 [57*]	USA	25	63.2 (11.4)	96%	Clinics and convenient	Alzheimer's Disease	[58*, 59*]

## Results

The selection process is displayed in Fig. 1. Sixty-three studies reported using ILMs among informal dementia caregivers. These studies were separated into two groups. Fifteen studies only used ILMs for descriptive purposes (IL data only for description, e.g., sleep diaries or activity recording) and were therefore excluded. The 48 remaining studies used ILMs to study psychological constructs and were integrated into the present review.

Several studies were published using the same dataset (See Table 1). For 48 published studies, it appears that there were only 22 different datasets that were included ( 1 to 14 publications per dataset). Thirteen datasets were used for only one publication. Consequently, 35 of the included publications were based on only 9 datasets.

### Objectives of the studies

Most studies used ILMs with an observational objective, to measure variables once or multiple times a day (Table 2). While most of these studies used ILMs as measurement (k = 17, i.e., using ILMs in observational designs), some also used them in conjunction with an intervention (k = 4, e.g., to track changes during a self-help intervention). The last study used ILMs as an intervention tool, as ILMs were used as a reflexive tool to monitor feelings, self-esteem, and well-being to increase well-being [14*].

**Table 2:** Summary of the designs of the included studies

Main reference	Purpose	Tool	Duration	Frequency	Main focus	#Beeps	ILMs CG-focused	ILMs Care-focused	Compliance
Bartels et al., 2020 [14*]	Intervention tool	App	3 days/week for 6 or 8 weeks	10	Momentary	180 or 240	Affect (PA and NA), Physical well-being, Self-esteem Activity at the moment, Activity-related stress		73–76%
Fauth et al., 2006 [20*]	Measurement	Diary	14 times in 90 days	One	Retrospective	14		Memory and Behavior problems and stress caused by it	n.m
Fonareva et al., 2012 [21*]	Measurement	PDA	1	4	Momentary	4	Emotional state (stressed/calm), Fatigue (Sleepy), Coping, Mindfulness, Situational demands		85%
Goodridge et al. 2021 [22*]	With intervention	App	84	One	Retrospective	84	Well-being (one item)		n.m
Jain et al., 2014 [23*]	With intervention	Diary	56	When meditating	Momentary	56	"Feeling state" (bad to good)		n.m
Jayalath et al., 2016 [24*]	Measurement	Diary	7	Event contingent	n.m	n.a		Dementia-related problems & caused distress	n.m
Koerner & Shirai, 2012 [25*]	Measurement	Diary	8	Once (pm)	Retrospective	8	Depressive symptoms, Subjective burden, Physical health, Non-care stressors	Caregiving tasks, Family disagreement regarding care, Memory and Behavior problems	98%
Konnert et al., 2017 [29*]	Measurement	Phone call	14	Once (pm)	Retrospective	14	Affect (PA & NA)	Daily conflict with healthcare professionals	98%
Liu et al., 2021 [30*]	With intervention	Phone call	8	Once (pm)	Retrospective	8	Depression, Affect, Sleep quality, Non-care stressors, Positive events, (Saliva samples), Body pain,	Use of day service, Care stressors	98%
MaloneBeach et al., 1995 [44*]	Measurement	Diary	14	One (pm)	Retrospective	14	Affect (PA & NA)	Caregiving activities, Dementia-related problematic behaviors	n.m
Mather et al., 2022 [45*]	Measurement	Phone call	8	One*	Retrospective	8	Affect (PA & NA) Sleep quality Anticipation of night sleep Daily stress Intensity of physical activity	CR sleep quantity CR Affect (PA & NA)	n.m
McCrae et al., 2016 [47*]	Measurement	Diary	7	One (am)	Momentary	7	Affect (PA & NA), Sleep time & quality (+ actigraphy for sleep),		n.m
Monin et al., 2017 [48*]	Measurement	Palm Pilots	8	5	Momentary	40	Affect (PA & NA)	Caregiving activity Time spent caregiving, Perception of partner's response to help	65%
Pickering et al., 2020 [50*]	Measurement	Email	21	2	Retrospective	42	Presence of self or social activities Receipt of instrumental support	Abusive and neglectful behaviors Caregiving stress Behavioral symptoms of dementia Disruption of routine Hours spent together	n.m
Pickering et al., 2022 [51*]	Measurement	Email & PIVRS	2 periods of 21 days over 18 months	1	Retrospective	42	(In)Formal support	Abusive and neglectful behaviors Stress from behavioral symptoms of dementia	88%
Pihet et al., 2017 [4*]	Measurement	Tablet	14	One (pm)	Retrospective	14	Subjective Burden, Psychological distress, Positive affect, Self-efficacy	CR problems and caused cg distress, Relationship quality	57%
Potts et al., 2020 [52*]	With intervention	iPad	84	When using app	n.m	n.a		Mutuality	n.m
Rullier et al., 2014 [53*]	Measurement	Phone call	4	5	Momentary	20	Current activity, Physical environment, Social company, Well-being, Sadness, Loneliness, Anxiety, Tiredness		87%
Ryuno et al., 2021 [54*]	Measurement	Diary	56	One (pm)	Retrospective	56	Affect (PA and NA) Subjective burden Actigraphy for sleep		62%
Savla et al., 2013 [55*]	Measurement	Phone call	7	Once (pm)	Retrospective	7	Physical symptoms, Affect (PA and NA), Competing demands, Leisure time activities, Non-care stressors, (Saliva sample)	Memory and behavioral problems, Marital interactions	100%
van Knippenberg, de Vugt, Ponds, Verhey, et al., 2018 [16*]	Measurement	App	6	10	Momentary	60	Affect (PA), Event-related stress, Activity-related stress		82%
Zawadzki et al., 2021 [57*]	Measurement	Diary	14	4	Retrospective	56	Affect (PA & NA), Leisure satisfaction; Activities performed & enjoyment from it		89%

*CG* Caregivers. *CR *Care-Recipients. *PA *& *NA* Positive and Negative Affects. *PIVRS* Phone Interactive Voice Response System

^*^ = Mather al. (2022) [45*] measured each variable once a day but collected sleep quality via a morning phone call and the other variables in the evening

It also appears that when researchers used ILMs as observational tools, their objectives differed. Most studies used it to explore associations between variables (e.g., associations between stress and mood), but 2 studies used ILMs to compare data collection methods, by confronting retrospective data collection with ILMs. These two studies respectively focused on comparing perceived stress fatigue, coping, and situational demands with an ILM and in a research setting [21*], and care-recipient’s problems using a daily diary vs in a weekly verbal report [24*].

Elements regarding the sampling focus (momentary vs retrospective) and the sampling frequency (i.e., the number of measurements per day) and duration (i.e., number of days) are displayed in Supplementary Materials 3.

### Implementation of ILMs

Regarding the different methods used to gather ILMs, most studies used diaries (k = 8) or telephone interviews (k = 5) (see Table 2). The others used a provided device (k = 4, e.g., a tablet or a PalmPilot), an app on the caregiver’s device (k = 3), or a complementary use of questionnaires sent by email or answered through a Phone Interactive Voice Response System (k = 2). Studies published before 2017 relied mostly on diaries and telephone interviews, whereas studies published since 2017 used a larger set of tools, which reflects a recent evolution in media used in ILMs. The data collection methods seem to evolve, notably through the inclusion of more digital methods.

Most studies focused on self-report for their measurement by collecting data from the caregiver alone. As such, all the self-reported variables were reported by the caregiver, whether it was regarding the stress they faced or their well-being. In addition to these self-report measures, some studies also included physiological measurements (e.g., cortisol alpha-amylase, and actigraphy to measure sleep. The caregiver report also included variables related to the care-recipient (e.g., mood, sleep quality, and memory and behavioral problems). There was no instance of dyadic data collection.

Studies did not report significant implementation problems and had high response rates (see Supplementary Materials 3 for a comparison of response rates based on the method used).

### Explored variables

The variables explored in the included studies mainly focused on the caregiver and the care(-recipient), as shown in Table 2. For the caregiver, studies explored their well-being, with measures of mood or affect, subjective burden, as well as physical well-being, body pain, depression, psychological distress, loneliness, and sleep quality. Additional measures focused on stress-related variables not specific to care, such as overall daily stress or event-related stress. Other psychosocial constructs were also investigated such as self-efficacy, focus on the present (“mindfulness”), social activities, and leisure or self-care activities.

The care(-recipients)-related variables mainly focused on the stress that providing care could represent in different forms: care-recipient’s memory and behavior-related problems, care-recipient’s sleep quantity and mood, caregiving tasks, and activities performed, disrupted routines, conflictual interactions with health-care professionals, and family disagreement regarding care. A few studies also had a particular focus on relational aspects with the care-recipient, such as marital interactions, mutuality, or relationship quality. Two recent studies also explored the violent and neglectful behaviors caregivers could have toward the care-recipient [50*, 51*].

### Measures and sampling used

The questionnaires used a wide variety of sources (see Table 3). Some were based on using full-validated scales adapted to the timeframe investigated whereas others used one or more items from existing scales. When items were to be selected from existing scales, they were selected for various motives such as being used in previous studies, selecting only clearly understandable items, or findings from previous studies guiding the choice. Some studies did not justify the choice of items. The authors also created new items (see Table 3). The majority of authors did not mention where the items came from, but some reported using guidelines for designing ILMs tools and researchers’ knowledge about the topic [15*]. Finally, some studies also used coding of open-ended questions, notably for the care-recipient’s behavioral problems, with made-up quantitative measures [24*] or caregiving activities [44*]. All these elements contribute to showing that there was no explicit or consistent rationale behind the item choice or creation, which questions the validity of the included measurements.

**Table 3:** Measures included in studies

	Variable investigated (Name of scales)	Studies exploring these variables
Full-validated scales	Subjective burden (ZBI)	Koerner & Shirai, 2012; Ryuno et al., 2021 [25*, 54*]
	Positive and negative affect (PANAS)	Bartels et al., 2020; Konnert et al., 2017; Liu et al., 2018; Mather et al., 2022; Ryuno et al., 2021; Zawadzki et al., 2021 [14*, 29*, 39*, 45*, 54*, 57*]
	Depression (HSCL & NSPDS)	Koerner & Shirai, 2012; Liu et al., 2018 [25*, 39*]
	Pleasantness of activities (PES-AD)	Zawadzki et al., 2021 [57*]
	Abusive behaviors (CTS2)	Pickering et al., 2020 [50*]
	Daily stress (DISE)	Liu et al., 2018; Akerstedt, 2010; Savla et al., 2013 [39*, 46*, 55*]
	Physical health symptoms (Checklist)	Koerner & Shirai, 2012 [25*]
	Family disagreement (Pearlin’s scale)	Koerner & Shirai, 2012 [25*]
Adapted versions	Subjective burden (ZBI)	Pihet et al., 2017 [4*]
	Care-recipient’s memory and behavioral problems (DRB)	Fauth et al., 2006; Liu et al., 2018; Pihet et al., 2017; Savla et al., 2013 [4*, 20*, 39*, 55*]
	Psychological Distress (Ilfeld’s scale)	Pihet et al., 2017 [4*]
	Mutuality	Potts et al., 2020 [52*]
	Positive and negative affect (PANAS)	McCrae et al., 2016 [47*]
	Sense of Competence (SSCQ)	van Knippenberg et al., 2017 [17*]
Created measurements	Emotional state or affect	Jain et al., 2014; Monin et al., 2017 [23*, 48*]
	Well-being	Goodridge et al., 2021 [22*]
	Neglectful behaviors	Pickering et al., 2020 [50*]
	Helping time and perception of partner’s response to help	Monin et al., 2017 [48*]
	Competing demands or leisure activities	Savla et al., 2013 [55*]
	Caring activities	MaloneBeach et al., 1995; Monin et al., 2017 [44*, 48*]
	Conflict with healthcare professionals	Konnert et al., 2017 [29*]

Notes: *ZBI* Zarit Burden Interview. *PANAS* Positive and Negative Affect Schedule. *HSCL* Hopkins Symptom Checklist. *NSPDS* Non-Specific Psychological Distress Scale. *PED-AD* Pleasant Events Schedule-AD. *CTS2* Revised Conflict Tactics Scale. *DISE* Daily Inventory of Stressful Events. *DRB* Daily Record of Behavior. *SSCQ* Short Sense of Competence Questionnaire

Studies used the most common designs of ILM studies [60] (see Table 2 and Table S1): daily diary (once a day), experience sampling (from 2 to 10 times per day), and event-contingent, while more rarely using burst designs (succession of several separate dense measurements). Variables explored in daily diary and experience sampling studies were slightly different. In daily diary studies, two sets of studies were identified. The first focused on the associations between different kinds of stressors or resources and well-being (mood, well-being, physical health, strain), and the second explored the association between sleep quality and well-being. For experience sampling studies, except for one study on neglectful behaviors [50*], all studies explored mood (affective states) and different forms of stress, in addition to other variables such as current activity, social company, or self-esteem.

Event-contingent studies were more heterogeneous, as they focused on the behavioral problems of the care-recipient [24*], the emotional state before and after daily mindfulness sessions [23*], or the relational quality between the care-recipient and the caregiver when they were using an app to increase the care-recipient’s reminiscence.

Supplementary Material 3 presents a more comprehensive depiction of the elements related to study design (including a focus on the measurement focus, duration of the studies, and a comparative description of what studies measured based on these designs).

### Analytic designs

Most studies explored data with mixed models, referred to under different terms (e.g., multilevel modeling, hierarchical linear models, linear mixed models). Depending on their research questions, studies relied on person- or grand mean-based analyses. Two studies used forms of structural equations modeling (SEM) that consider the multilevel nature of the data, namely the dynamic SEM [61*] and the multilevel SEM [45*].

Only a few studies included time in their analyses, such as the day of the week or time trends. Two studies performed longitudinal analysis (using growth curve models) to explore the stability of indicators over time [20*, 53*]. Three studies explored lagged associations (i.e., associations between variables at T-1 and T0) between two days [34*, 61*] or between consecutive moments of the day [57*]. Other studies stated controlling for reverse causation using lagged interval, without interpreting it [48*, 49*].

A final set of studies did not account for the nested variability of the data and aggregated the scores. In these studies, the individual scores were averaged for each individual, without mentioning if a method was used to take into account the variability due to multiple measurements.

### Results of the studies

The third objective of the present review was to synthesize the results of the studies. One of the main observations shared among studies was the important intra-individual variability in the explored variables, supporting the importance of using ILM designs in informal care. Whether it was regarding well-being, affect, or stress, the larger part of the variability found was intra-individual. The occurrence of problematic behaviors from the care-recipient was particularly subject to intra-individual variability, between 55 and 62% [4*, 20*, 26*]. For well-being indicators, variability was between 27 and 63% for burden, 37% to 43% for depression and 66% for psychological distress, 34% for physical health symptoms, 65% within body pain, and 69% for sleep quality [4*, 26*, 34*, 35*, 47*]. One study reported an intra-variability of 33% for positive emotions and 44% for negative emotions [47*] and another showed 49% of intra-individual variability in anger [35*]. Relationship quality with the care-recipient also widely varied between days (59%, 4), as well as abusive (65%) and neglectful (60%) behaviors toward them [50*]. Taken together, these elements show an intra-individual variability ranging from 27 to 69%.

Regardless of their designs, the results of the different studies converge on different aspects. Overall, more stress during the day was associated with poorer well-being. More specifically, care-related stress (caregiving tasks, behavioral problems) was associated with more distress (burden, depression, caregiving stress) [27*, 61*], more negative and less positive emotions [16*, 44*, 49*, 56*], and more body pain [34*]. Poor care-recipient’s sleep and high care-recipient’s negative emotions were associated with higher negative affect [30*, 45*, 46*]. Distress related to memory and behavior problems was associated with burden [4*]. Conflicts with the caring staff were also a source of lower positive and higher negative emotions [29*]. Other forms of stress, such as family disagreement, unpleasant interactions, or low relationship quality with the care-recipient were associated with lower well-being (higher burden, 4, more negative affect 38, higher depression and burden, 57). Non-care-related stressors, disturbed routines, and lower sleep quality were also associated with higher negative emotions [45*, 47*, 54*, 56*]. The association between stressors and well-being was also found with biological markers such as cortisol and alpha-amylase [39*, 55*, 56*], as well as between negative emotions and cortisol [35*]. One study also highlighted that caregivers’ behaviors that could stress the care-recipient lead to problematic behaviors the same and the next day [61*]. All these results show that higher stress leads to poorer well-being and that this stress can take many different forms (whether care-related, social, or individual).

Aside from the potential risk factors, several factors increased caregivers’ well-being. Pleasant, self-care, or leisure activities and enjoyment from them were associated with more positive affect and less negative affect [14*, 40*, 57*, 58*]. Sense of competence was associated with positive affect [17*]. Using adult day services was associated with more positive affect, lower stress levels, decrease in behavioral problems, improved sleep, less time spent with the care-recipient, and more positive experience [32*, 36*, 37*, 38*, 39*, 40*, 41*, 42*, 43*]. There was also less negative affect variability when using such services, which was associated with fewer daily stressors, greater care-related stressors, more positive events, and less-than-average dependency of the care-recipient.

Different variables influenced these associations, which provides more nuanced investigations. Coping strategies such as seeking distraction and seeking social support, fostering reassuring thoughts, as well as a high sense of competence and mastery diminished the association between stress during the day and negative affect [16*]. Women had a more important association between stressors and well-being than men, as well as caregivers with high neuroticism, low extroversion, or low conscientiousness [26*], or low level of socio-emotional support and high levels of familism [25*, 27*]. Relationship quality with the care-recipient has been shown to moderate the association between care-related stress and distress, with the notable exception of reality problem symptoms, whose effects are worsened when the relationship quality is higher [33*]. In addition, helping the care-recipient was associated with positive affect only when perceiving that it had a positive impact on the care-recipient [48*] and when there was an important interdependence with the care-recipient [49*]. Using adult day services buffers the impact of care-related stress (e.g., CR’s sleep problems) on well-being [30*]. Caregivers with higher burden benefitted more from leisure activities, with more important associations between leisure satisfaction and negative affect if high on burden [59*]. The benefit of sufficient sleep on well-being was shown to be particularly important for working caregivers, as opposed to those who were unemployed [54*]. One study also suggested that there could be different clusters of caregivers with differentiated associations between stress and well-being [44*]. The only study performed during the COVID-19 pandemic showed that there was no increase in abuse and neglect behaviors during that period [51*]. These insights suggest that coping, gender, personality, relationship quality with the care-recipient, cognitions, well-being, and use of respite care services modify associations between stress and well-being.

Finally, studies using ILMs to compare methods showed that ILMs were more accurate than other retrospective tools (e.g., lab report or oral retrospective recollection) for most of the investigated variables (i.e., stress and care-recipient’s behavioral, cognitive, emotional, and psychiatric problems), albeit not all of them (i.e., coping, mindfulness, situational demand, fatigue) [21*, 24*].

## Discussion

The present scoping review aimed at exploring the use of intensive longitudinal methods (ILMs) in the context of providing informal care to a relative with dementia. After the selection process, 48 studies published between 1995 and 2022 were identified. The 48 studies were based on 22 different datasets, with between 1 and 14 publications per dataset.

First, the included studies provided interesting results in the understanding of informal caregivers’ daily lives. One finding was to highlight the important intra-individual variability of the investigated variables. This variability was expected, as it was highlighted in other fields of research [62], but the magnitude of intra-individual variability was particularly striking, often close to 60%, suggesting that, from a clinical standpoint, speaking of “good and bad days” in informal care is a tangible reality supported empirically [4*]. This observation stands for the individual well-being of the caregiver, but also underscores the variability of the care-recipient’s symptomatology, as it had been highlighted in the patient-focused research [56*] and causing an important feeling of unpredictability, often reported in qualitative inquiries [63]. Taken together, these findings also strengthen the observation that cross-sectional measures only capture a fraction of the individual variability and encourage exploring the determinants of variability that seem to have been neglected for now. Understanding the causes of the variation for the explored variables would highlight why some individuals have higher or lower variations in their scores [64].

Alongside this heterogeneity, most of the included studies explored the association between stressors and well-being, whether it was to identify protection or risk factors or what could influence the association between stress and well-being. This naturally stems from the major reliance of these studies on stress and burden-based models (e.g., Pearlin’s model). Studies mostly showed that experiencing stress during the day was associated with a decrease in psychological well-being. As such, these investigations provide important insights but may fail to benefit from the advantages of the ILMs. Such advantages are to explore individual variability and perform in situ momentary assessments, notably to investigate how this stress was managed and what were its consequences later. Although demanding and complex, such a perspective would also benefit from including dyadic aspects, whether with the care-recipients or other family members [65]. In addition, emotions were only investigated as well-being outcomes. The variability of emotions was considered in light of the intraindividual variability. Still, there was little consideration of the dynamic of emotions and their flow over time [66], nor of the cause it the variability or their regulation, which is a domain where ILMs are particularly promising [67]. Studying emotions and their regulation is particularly important in the “emotional roller coaster” of informal caregiving, where both the care and the emotions it causes importantly varied from one day to the other [68]. Such inquiry should not overlook the investigation of causes and consequences of positive emotions, which are often overlooked in caregiving research [69].

Aside from exploring associations, ILMs were also used in accompanying an intervention. Most studies had no clear theoretical integration of these measures in the intervention [22*, 52*]. One of the main missing ways to implement ILMs was the longitudinal monitoring of the intervention, which would help to have a precise understanding of the processes involved over the course of the intervention. The use of ILMs would allow a better understanding of the trajectories of individuals following the intervention, and provide complementary information to regular pre/post measurements, as shown in other fields (e.g., [70]). Such an approach would be particularly relevant in *N *= 1 analyses to combine the quantitative follow-up with qualitative insights into the caregiver’s experience. In the long run, it could also inform us on evolutions throughout the different phases of dementia, e.g., by identifying how certain deteriorations influence daily life [71]. These trajectories could allow the exploration of moderating factors, identifying trajectories based on different moderators, such as relationship with the care-recipient or initial level of well-being.

Only one dataset used ILMs as an intervention tool, which nevertheless seems promising as the data collection can be a form of intervention, especially if augmented with a regular follow-up with a clinician [14*, 18*]. This approach is closer to what could be done in clinical settings, where the use of ILMs allows tailoring the interventions to the reality of each person [72]. While getting closer to their daily experience, it would also allow the caregivers to reflect upon their experience. As the results have shown in informal care [18*] and the general population [73], only using it as a reflexive tool does not seem to suffice to improve (or worsen) well-being but could contribute to identifying the point of attention for the clinicians and fuel the therapeutic process.

Regardless of the design and intention of each study, one of the striking results was also the absence of clear guidelines for the measurement tools used. It appears that most authors had to be creative in finding adequate measurements to answer their research question and used different techniques to do so. Authors created new tools, adapted long questionnaires, and selected some items of validated questionnaires, but few used tools that were already used in previous studies. This issue is not exclusive to the present review, as it was also pointed out in other fields [74, 75]. The necessity is therefore to use a more standardized approach of measurement in ILM studies, notably through the validation of new tools, but also through the record of items already created or used [76].

If the items interrogate the validity of the content, the analyses used also question what information is extracted from ILM data. It appears that most of the studies used a form of mixed-effect models to analyze their data [77]. This approach allows the consideration of the longitudinal nature of the data, with multiple measurements per individual. In the present review, researchers mainly used these analyses as they would with linear regression models for cross-sectional data, focusing on the sample’s mean. We would however benefit from expanding the use of person-centered analysis in these mixed-effect models, which relies on using the mean of every individual in the analysis (within person-centering) or means per specific groups (between-person centering) in opposition to the usual centering around the sample’s mean (grand centering) [78]. This would provide insights into informal caregivers’ difficulties and turning points, such as the individual “tolerance line” [79]. Interpreting the data would therefore focus on circumstances when caregivers are above or below their mean, which is particularly relevant when exploring stress, behaviors, and emotions.

Further on the analyses, there was little use of the temporality of the measurement, such as considering how one variable is influenced by variables from previous measurements (i.e., temporal dependence), which covers methods such as autoregressive and time-lag models [80]. Three studies applied statistical methods to explore such effect, which allowed them to understand how the investigated variables unfold over time, e.g., showing the influence of activity on positive affect later during the day, the lasting effect of adult services the next day, or the dynamic of stress over days [34*, 57*, 61*]. The other studies did not focus on temporality as they only investigated associations between variables of the same time point. Therefore, they may miss part of the potential benefits of using ILMs. Based on the included variables, time lag models may not have been applied in each of them, but it is in the design itself of the studies that this approach could have been implemented upfront. Therefore, these studies did not take into account potential temporal causality (or even temporal dependence), but also mutual influence and reciprocal interactions between stressors and well-being over time [67] that could also be taken into account through network analysis [81]. A better understanding of these dynamics and processes would also provide more information to design adequate interventions through more precise targeting of key processes.

### Limitations

The first limitation of the present review is the use of heterogeneous terminologies to define ILMs in the literature. Despite the existence of a set of already complex and partially overlapping terms used to characterize these studies (e.g., EMA, ESM, ILMs, diary studies), there was no systematic use of such terminologies in most of the included studies. Two consequences stem from that observation. First, the keywords used in the literature search were more extensive than in the protocol that was established for the present review. Second, and despite this extension, studies could have been missed due to the absence of the use of common terminologies.

A potential publication bias exists that only the studies with adequate compliance would have been published. However, as reporting is still far from systematic in ILM studies [82], studies with lower rates could still have been published, as illustrated by the fact that one-third of the dataset included in the present review did not report these rates. In addition to reporting the global compliance, there was little use of compliance threshold, where part of the sample is left out based on too low compliance, as only one study reported it [18*].

The present review would also have benefited from including studies from CINAHL to ensure the inclusion of available work in nursing studies.

## Conclusion

In the end, it appears that ILM is a feasible tool that has already yielded interesting results in informal care research, notably by highlighting individual variability and how daily stress can influence the caregiver’s well-being. Conclusions drawn from the present review however highlighted the possibility to exploit these tools even further, at different levels. As a fundamental tool to understand the daily experience of informal caregivers, it appears that what had been studied for now is only limited to a certain range of variables that do not address emotion and emotion regulation. Beyond that, the implied necessity is to explore the *dynamic* of informal caregivers’ daily lives, whether it is through the design of the study, the investigated variables, or the way data are analyzed with models that include time as a variable of interest (i.e., time-lag approaches). To do so, researchers should not hesitate in designing studies that would be shorter in time but more intensive with multiple momentary measurements during the day. While doing so, particular attention will have to be drawn to the use or development of validated tools to measure the variables of interest, as this area may appear as the Achilles heel of the field in the long run.

Overall, this research field in informal care is still in its infancy but opens new perspectives in having a better understanding of the daily life of informal caregivers, as a complement to retrospective-based studies. The insights provided by the published studies included in the present review will contribute to building a true exploration of the daily life challenges and resources informal caregivers experience.

## Supplementary Information

**Additional file 1.**

## Data Availability

All data generated or analyzed during this study are included in this published article (literature review based on published materials).
