# Supplementary material for: A scoping review of intensive longitudinal methods in informal caregivers of people with dementia
Source: BMC Geriatr. 2023 Jul 25;23:456. doi: 10.1186/s12877-023-04123-6 (PMC10367249; doi:10.1186/s12877-023-04123-6)
Supplement: Supplementary file 1 — Additional file 1. [file 12877_2023_4123_MOESM1_ESM.docx]

## Supplementary Materials (Appendixes)

## Supplementary Material 1. Preferred Reporting Items for Systematic reviews and Meta-Analyses extension for Scoping Reviews (PRISMA-ScR) Checklist

| **SECTION** | **ITEM** | **PRISMA-ScR CHECKLIST ITEM** | **REPORTED ON PAGE #** |
| --- | --- | --- | --- |
| **TITLE** | | | |
| Title | 1 | Identify the report as a scoping review. | 1 |
| **ABSTRACT** | | | |
| Structured summary | 2 | Provide a structured summary that includes (as applicable): background, objectives, eligibility criteria, sources of evidence, charting methods, results, and conclusions that relate to the review questions and objectives. | 2 |
| **INTRODUCTION** | | | |
| Rationale | 3 | Describe the rationale for the review in the context of what is already known. Explain why the review questions/objectives lend themselves to a scoping review approach. | 3-4 |
| Objectives | 4 | Provide an explicit statement of the questions and objectives being addressed with reference to their key elements (e.g., population or participants, concepts, and context) or other relevant key elements used to conceptualize the review questions and/or objectives. | 4 |
| **METHODS** | | | |
| Protocol and registration | 5 | Indicate whether a review protocol exists; state if and where it can be accessed (e.g., a Web address); and if available, provide registration information, including the registration number. | 4 |
| Eligibility criteria | 6 | Specify characteristics of the sources of evidence used as eligibility criteria (e.g., years considered, language, and publication status), and provide a rationale. | 4-5 |
| Information sources* | 7 | Describe all information sources in the search (e.g., databases with dates of coverage and contact with authors to identify additional sources), as well as the date the most recent search was executed. | 5 |
| Search | 8 | Present the full electronic search strategy for at least 1 database, including any limits used, such that it could be repeated. | 5 |
| Selection of sources of evidence† | 9 | State the process for selecting sources of evidence (i.e., screening and eligibility) included in the scoping review. | 5 |
| Data charting process‡ | 10 | Describe the methods of charting data from the included sources of evidence (e.g., calibrated forms or forms that have been tested by the team before their use, and whether data charting was done independently or in duplicate) and any processes for obtaining and confirming data from investigators. | 5-6 |
| Data items | 11 | List and define all variables for which data were sought and any assumptions and simplifications made. | 5-6 |
| Critical appraisal of individual sources of evidence§ | 12 | If done, provide a rationale for conducting a critical appraisal of included sources of evidence; describe the methods used and how this information was used in any data synthesis (if appropriate). | n.a. |
| Synthesis of results | 13 | Describe the methods of handling and summarizing the data that were charted. | 6 |
| **RESULTS** | | | |
| Selection of sources of evidence | 14 | Give numbers of sources of evidence screened, assessed for eligibility, and included in the review, with reasons for exclusions at each stage, ideally using a flow diagram. | 9 |
| Characteristics of sources of evidence | 15 | For each source of evidence, present characteristics for which data were charted and provide the citations. | 9 |
| Critical appraisal within sources of evidence | 16 | If done, present data on critical appraisal of included sources of evidence (see item 12). | n.a. |
| Results of individual sources of evidence | 17 | For each included source of evidence, present the relevant data that were charted that relate to the review questions and objectives. | 10-21 |
| Synthesis of results | 18 | Summarize and/or present the charting results as they relate to the review questions and objectives. | 10-21 |
| **DISCUSSION** | | | |
| Summary of evidence | 19 | Summarize the main results (including an overview of concepts, themes, and types of evidence available), link to the review questions and objectives, and consider the relevance to key groups. | 21-24 |
| Limitations | 20 | Discuss the limitations of the scoping review process. | 24-25 |
| Conclusions | 21 | Provide a general interpretation of the results with respect to the review questions and objectives, as well as potential implications and/or next steps. | 25-26 |
| **FUNDING** | | | |
| Funding | 22 | Describe sources of funding for the included sources of evidence, as well as sources of funding for the scoping review. Describe the role of the funders of the scoping review. | Submission portal |

## Supplementary Material 2. Keywords used to retrieve studies.

Different keywords were used to retrieve the studies. These keywords were of three complementary sets addressing (a) informal care, (b) dementia or related diseases, and (c) intensive longitudinal methods. These keywords were mainly based on the authors' experience in the field, as well as on keywords from studies retrieved in an exploratory screening performed in October 2020. Because ILM covers a large range of methods and measurements, different keywords were used (e.g., Ecological Momentary Assessment, Daily Diary, Experience Sampling Method, Daily affect, Daily Stress). Truncations were used to ensure that all potential variations were included. The exhaustive list of keywords used is presented below.

| Caregivers | Disease | ILM |
| --- | --- | --- |
| ("informal car*")  OR ("family car*")  OR ("Carer*")  OR ("Caregiv*")  OR ("Partner*")  OR (“Spouse*")  OR (“Parent*”) | ("Dementia")  OR ("Alzheimer")  OR (“Huntington")  OR ("Parkinson")  OR (“Neurocognitive Disorder*”)  OR ("Neurodegenerative Disease*")  OR ("Nervous System Disorder*")  OR ("Neurodegeneration")  OR (“Cognitive ag*ing”)  OR (“Cognitive impairment”)  OR (“neuromuscular disease*”)  OR (“Fronto-temporal”) | ("ESM")  OR ("EMA")  OR ("Experience sampling method*")  OR (“Experience sampling*”)  OR ("Ecological momentary Assessment*")  OR ("Ambulatory assessment*")  OR ("Momentary assessment*")  OR (“Intensive Longitudinal Data*”)  OR (“Intensive Longitudinal Method*”)  OR (“time series data*”)  OR (“daily diar*”)  OR (“Real-time data”)  OR (“Quantified Self”)  OR (“MHealth”)  OR (“Daily mood”)  OR (“intra-individual”)  OR (“daily cortisol”)  OR (“daily stress*”)  OR (“daily emotion*”)  OR (“daily affect*”)  OR (“daily fluctuation*”)  OR (“daily telephone”)  OR (“24-hour*”)  OR (“daily sleep”)) |

## Supplementary materials 3: Sampling frequency, duration of studies, and feasibility.

***Sampling frequency***

An element that was not mentioned in the main text was the fact that studies can also be divided regarding the main focus of their measurement (1): momentary (at the moment) or retrospective (during a period of time). This distinction, although not critical in the appraisal of the current literature, adds a layer of understanding of the included studies, notably by bridging it with the ILM design.

Daily diary studies explored different variables by using one fixed measurement per day, as shown in Table 2. This sole measurement was mostly performed in the evening (2–6), and less often in the morning, only when sleep-related variables were included (7,8). One study combined these two moments, with one data collection in the morning for sleep quality, and one in the evening for affect and daily stress (9). Experience sampling studies used a mix of fixed and random interval for their questionnaires, but even random questionnaires occurred during fixed time windows, which was referred to as semi-random schedules assessments (e.g., random prompt between 9 and 11, 10).

Variables explored in daily diary and experience sampling studies were slightly different. In daily diary studies, two sets of studies were identified. The first focused on the associations between different kinds of stressors or resources and well-being (mood, well-being, physical health, strain) (2–6,11), and the second explored the association between sleep quality and well-being (7,8,12). For experience sampling studies, with the exception of one study on neglectful behaviors (13), all studies explored mood (affective states) and different forms of stress, in addition to other variables such as current activity, social company, or self-esteem (9,10,14–18).

Event-contingent studies were more heterogeneous, as they focused on behavioral problems of the care-recipient (19), emotional states before and after daily mindfulness sessions (20), or relational quality between the care-recipient and the caregiver when they were using an app to increase the recipient’s reminiscence (21).

Regarding the focus of the questions asked, it appears that most studies used retrospective questionnaires (k = 12, i.e., people had to answer regarding how they felt or what occurred since the last questionnaire), and slightly fewer used momentary questionnaires (k = 7, i.e., answer *in situ,* regarding their current feelings at the moment of the questionnaire). Two of the three event-contingent studies were unclear regarding the focus of the questions asked. Daily diary studies were mostly relying on retrospective measures (10/2, 83%), whereas experience sampling studies mainly focused on momentary measurements (5/2; 71%). As for the design, there was no clear difference in included variables between the two (see Table S1), as there were occurrences of mood and stress in both kinds of studies whether they were daily diaries or experience sampling. Studies with a retrospective focus however most often included more stable constructs such as subjective burden, physical symptoms, and occurrence of recipient’s memory and behavioral problems.

| Table S1. Summary of the categories of designs used. | | | | | |
| --- | --- | --- | --- | --- | --- |
| Variables |  | Daily diary  k = 12 | Experience sampling  k = 7 | Event-contingent  k = 3 | Total  = 22 |
| Purpose |  |  |  |  |  |
|  | Measurement | 10 (83%) | 6 (86%) | 1 (33%) | 17 (77%) |
|  | Intervention tool | 0 (0%) | 1 (14%) | 0 (0%) | 1 (5%) |
|  | With intervention | 2 (17%) | 0 (0%) | 2 (67%) | 4 (18%) |
| Tool |  |  |  |  |  |
|  | Diary | 5 (42%) | 1 (14%) | 2 (67%) | 8 (36%) |
|  | Telephone | 4 (33%) | 1 (14%) | 0 (0%) | 5 (23%) |
|  | Provided device | 1 (8%) | 2 (29%) | 1 (33%) | 4 (18%) |
|  | App | 1 (8%) | 2 (29%) | 0 (0%) | 3 (14%) |
|  | Email | 0 (0%) | 1 (14%) | 0 (0%) | 1 (5%) |
|  | Email & PIVRS | 1 (8%) | 0 (0%) | 0 (0%) | 1 (5%) |
| Mean duration |  | 23 [7-84]* | 11.4 [1 - 24] | 49 [7 - 84] |  |
| Mean frequency |  | 1 | 5.7 [2 - 10] | n.a. |  |
| CG Variables | Well-Being | Affect (PA & NA), Physical health, Subjective burden, Depression, Psychological distress, Sleep quality and quantity, well-being, body pain | Affect (PA & NA), Physical well-being, Fatigue, Well-being, Loneliness, Anxiety | "Feeling state" (bad to good) |  |
|  | Stress | Daily stress, Non-care stressors, Positive events | Event-related stress, Activity-related stress |  |  |
|  | Psychosocial | Intensity of physical activity, self-efficacy, leisure time activities, (In)formal support | Current activity (and enjoyment), self or social activities, physical environment, social company, instrumental support, coping, mindfulness, Leisure satisfaction, Self-esteem |  |  |
|  | Physiological | Saliva samples, Actigraphy (sleep) |  |  |  |
| CR Variables | Stress | Memory and Behavior problems, caregiving activities, care stressors, CR sleep quantity, CR Affect (PA & NA) | Memory and Behavior problems and stress caused by it, Disruption of routine, Caregiving activity, caregiving stress, time spent caregiving | Memory and Behavior problems, Cause distress |  |
|  | Relational | Relationship quality, Marital interactions, abusive and neglectful behaviors | Perception of partner's response  Abusive and neglectful behaviors | Mutuality |  |
|  | Social | Conflict with professionals, Family disagreement, Use of day service |  |  |  |
| Notes. CG = Caregivers. CR = Care-Recipients. PA & NA = Positive and Negative Affects. PIVRS = Phone Interactive Voice Response System.  * As Fauth et al., (2006) and Pickering et al., (2022) used burst designs, only the number of measurement days was included (n = 14, n = 42). | | | | | |

### Duration of the studies

Overall, the duration of the daily diary and experience sampling studies importantly varied, from one to more than 84 days, and the sampling frequency of measures per day varied from one to 10 (see Table 2). As illustrated in Figure S1, studies with daily diary design mainly lasted one week (7 or 8 days) or two (14 days), with the exception of two studies that lasted 56 and 84 days (12,22). For experience sampling studies, 57% of studies had 4 or 5 measurement per day regardless of the duration. The other studies was constituted of two studies with 10 measurements per day for 6 and 56 days, and one with 2 measurements per day for 21 days. Only two studies presented a “burst design” (11,23), which refers to having several ILMs in short, distinct periods of time (e.g., 3 consecutive days every 10 days, Sliwinski, 2008). Altogether, the majority of daily diary or experience sampling studies (13/19, 68%) are in the same area, as they lasted for a maximum of 14 days, with a maximum of 5 measurements per day, regardless of their design. Event-contingent studies lasted respectively 7, 56, and 84 days (19–21).

Figure S1.

Figure. S1. Duration and sampling frequency of the included studies.

Note: Studies using an event-contingent design were not included in the graph as the sampling frequency is based on the number of events and not standardized frequency. Studies using burst designs were only accounted for the number of measurement days.

### Feasibility of ILMs

The last objective was to synthetize the feasibility of ILMs. Response rates were not always reported but for those that reported it (k = 14), the rates appeared high, with an average of 83% (SD = 14.0%). Studies using telephone interviews had the highest response rates (M = 96%, SD = 5.9%) (4–6,17) in comparison to studies with devices (M = 73%, SD = 11.8%) (2,10,14,16,18). The three studies using diaries also had a high response rate but with an important variability (M = 83%, SD = 18.7%) (12,24,25), and the two studies using a combination of emails and automatic telephone answers had a response rate of 85% (SD = 4,2%) (23,26). Eight sets of studies did not report the compliance rate at all. In addition, studies rarely reported their attrition rates, but when they did, it was very low, mostly below 5% (4,16,27,28). Some authors however modified the duration of the study when there was a change in individual caring situations (e.g., 12) or observed that caregivers adapted their response pattern (e.g., they did not answer on the days where they did not meet the care-recipient, 2). In one case, authors listed the *a priori* motives for not participating in the study of using ILMs as an intervention tool, and the burdensome aspect, time-consuming, and – to a lower extent – unfamiliarity with technological devices were among the most important cited motives for not participating (18,29).

Most studies did not report particular issues, including the use of electronic devices when it was the case (e.g., 2,14). On the contrary, studies using questionnaires to gather the participants’ feedback were very positive, with participants reporting easy completion of the different questionnaires and maintaining their participation (e.g., 2,4,10,17). Several studies however acknowledged a potential (self-)selection bias of the included caregivers and that their sample size would benefit from being larger and more representative (e.g., 9,12,15,30).

## Elements of discussion regarding the intensity of measurement, duration of studies, and feasibility of studies.

### A feasible approach

To begin with, it appears that, based on the included studies, using ILMs with informal caregivers is feasible and leads to an important set of information, despite the expected difficulties to use such methods in the older population. Compliance rates (when reported) were on average high, regardless of the method used, and the included feasibility studies have shown that participants report only a few issues in participating in ILMs studies. We can only recommend that authors systematically report their compliance and drop-out rates, as well as difficulties encountered, in order to better inform future research. Nevertheless, this observation is promising and echoes previous studies that have also shown that early stages dementia patients are capable of using (digital) tools for ILMs (31). These two observations combined leave an open horizon for the implementation of ILMs in dementia care research.

### Beyond the balance between intensity and duration

In ILM research, the duration and sampling frequency of studies is always a difficult balance between the burden for the participant and conceptual relevance (32). In the present case, most included studies relied on designs using between 1 to 5 measurements a day for a duration of 7 to 14 days. When looking at the graph displaying the duration and intensity of the measurements (Figure S1), we can only consider three gaps: there are only a few ILM studies longer than 14 days, using burst designs (i.e., multiple measurements phases over a longer period of time), or exploiting ILMs multiple times a day. The latter is an opportunity to explore the *dynamics* rather than focusing on associations between variables (33). As it has been shown in other fields, studies could rely on designs using up to 10 measurements per day for a few days and still display good compliance rates (34,35). The duration of the study does not necessarily have to last more than a few days if the measurements is adequately designed to capture the variability of the explored variables. The sampling frequency must be a compromise between the participant’s burden and the expected variability of these variables. The use of burst designs could also offer interesting perspectives, especially in the context of accompanying interventions (36). By having several dense but short ILMs sessions, the information gathered would provide different pictures to compare, closer to the caregiver’s experience than the use of retrospective questionnaires (37). In such designs, informal caregivers would participate in multiple sessions of EMA, before and after the intervention, which could also provide better validity of the results of clinical studies and trials (38). Regardless of their designs, one of the specificities of this field is that informal caregivers may not provide care every day, as there is a large range of intensity of care (39). Researchers should therefore be mindful in targeting days that fit the purpose of their study, whether including days when caregivers may or not actually provide care to the recipient, or a mix of the two.

### Measuring then or now

Alongside this observation was the focus of the measurement. Most of the experience sampling studies relied on momentary measures, which focus on the current state of the person at the moment of filling out the questionnaire. This is recognized as one of the true benefits of using ILMs, as it is the closest-to-moment self-report measurement possible, in opposition to retrospective ones that maintain a certain risk of bias (1,40). However, there was no clear difference between studies based on that focus, as the variables investigated did not importantly vary (e.g., stress or emotions were measured using both momentary and retrospective measures). Additionally, experience sampling studies relied more on momentary assessment, but some of them rather used retrospective measures, even if they investigated a comparable set of variables. There was therefore no clear distinction between the two designs. Future studies should therefore more explicitly take into account the focus of their measurement to increase transparency but also to consider how it affects their results.

References

1. Bolger N, Laurenceau JP. Intensive longitudinal methods: An introduction to diary and experience sampling research. Guilford Press; 2013.

2. Pihet S, Passini CM, Eicher M. Good and bad days: Fluctuations in the burden of informal dementia caregivers, an experience sampling study. Nurs Res. 2017;66(6):421–31.

3. MaloneBeach EE, Zarit SH, Farbman D. Variability in daily events and mood of family caregivers to cognitively impaired elders. Int J Aging Hum Dev. 1995;41(2):151–67.

4. Konnert C, Speirs C, Mori C. Conflict between Family Caregivers and Staff in Nursing Homes: Feasibility of the Daily Diary Method. Clin Gerontol. 2017 Dec;40(5):352–61.

5. Liu Y, Almeida DM, Rovine MJ, Zarit SH. Modeling cortisol daily rhythms of family caregivers of individuals with dementia: Daily stressors and adult day services use. J Gerontol B Psychol Sci Soc Sci. 2018;73(3):457–67.

6. Savla J, Granger DA, Roberto KA, Davey A, Blieszner R, Gwazdauskas F. Cortisol, alpha amylase, and daily stressors in spouses of persons with mild cognitive impairment. Psychol Aging. 2013;28(3):666–79.

7. McCrae CS, Dzierzewski JM, McNamara JPH, Vatthauer KE, Roth AJ, Rowe MA. Changes in Sleep Predict Changes in Affect in Older Caregivers of Individuals with Alzheimer’s Dementia: A Multilevel Model Approach. J Gerontol - Ser B Psychol Sci Soc Sci. 2016;71(3):458–62.

8. Akerstedt AM. Sleep Disturbances in Alzheimer’s Disease and Caregiver Mood: A Diary Study. Gerontologist. 2010 Oct;50:231–231.

9. Mather MA, Laws HB, Dixon JS, Ready RE, Akerstedt AM. Sleep Behaviors in Persons With Alzheimer’s Disease: Associations With Caregiver Sleep and Affect. J Appl Gerontol. 2022 Jan;41(1):295–305.

10. Fonareva I, Amen AM, Ellingson RM, Oken BS. Differences in stress-related ratings between research center and home environments in dementia caregivers using ecological momentary assessment. Int Psychogeriatr. 2012;24(1):90–8.

11. Fauth EB, Zarit SH, Femia EE, Hofer SM, Stephens MAP. Behavioral and psychological symptoms of dementia and caregivers’ stress appraisals: Intra-individual stability and change over short-term observations. Aging Ment Health. 2006 Nov 1;10(6):563–73.

12. Ryuno H, Yamaguchi Y, Greiner C. Effect of Employment Status on the Association Among Sleep, Care Burden, and Negative Affect in Family Caregivers. J Geriatr Psychiatry Neurol. 2021 Nov;34(6):574–81.

13. Pickering CEZ, Yefimova M, Maxwell C, Puga F, Sullivan T. Daily Context for Abusive and Neglectful Behavior in Family Caregiving for Dementia. The Gerontologist. 2020 Apr 2;60(3):483–93.

14. Bartels SL, van Knippenberg RJM, Viechtbauer W, Simons CJP, Ponds RW, Myin-Germeys I, et al. Intervention mechanisms of an experience sampling intervention for spousal carers of people with dementia: a secondary analysis using momentary data. Aging Ment Health. 2020;26(2):294–304.

15. Zawadzki MJ, Small AK, Mausbach BT. An upward cycle: examining bidirectional relationships between everyday activities and momentary affective well-being in caregivers. J Posit Psychol. 2021;

16. Monin JK, Poulin MJ, Brown SL, Langa KM. Spouses’ daily feelings of appreciation and self-reported well-being. Health Psychol. 2017;36(12):1135–9.

17. Rullier L, Atzeni T, Husky M, Bouisson J, Dartigues JF, Swendsen J, et al. Daily life functioning of community‐dwelling elderly couples: An investigation of the feasibility and validity of ecological momentary assessment. Int J Methods Psychiatr Res. 2014;23(2):208–16.

18. van Knippenberg RJ, de Vugt ME, Ponds RW, Myin-Germeys I, Verhey FRJ. An experience sampling method intervention for dementia caregivers: Results of a randomized controlled trial. Am J Geriatr Psychiatry. 2018;26(12):1231–43.

19. Jayalath D, Ashaye K, Kvavilashvili L. Carers’ diaries in dementia: Is there a role in clinical practice? Alzheimers Dement Amst. 2016;4:94–8.

20. Jain FA, Nazarian N, Lavretsky H. Feasibility of central meditation and imagery therapy for dementia caregivers. Int J Geriatr Psychiatry. 2014;29(8):870–6.

21. Potts C, Bond R, Ryan A, Mulvenna M, McCauley C, Laird E, et al. Ecological Momentary Assessment Within a Digital Health Intervention for Reminiscence in Persons With Dementia and Caregivers: User Engagement Study. JMIR Mhealth Uhealth. 2020 Jul 6;8(7):e17120.

22. Goodridge D, Reis N, Neiser J, Haubrich T, Westberg B, Erickson-Lumb L, et al. An App-Based Mindfulness-Based Self-compassion Program to Support Caregivers of People With Dementia: Participatory Feasibility Study. JMIR Aging. 2021 Nov 26;4(4):e28652.

23. Pickering CEZ, Maxwell CD, Yefimova M, Wang D, Puga F, Sullivan T. Early Stages of COVID-19 Pandemic Had No Discernable Impact on Risk of Elder Abuse and Neglect Among Dementia Family Caregivers: A Daily Diary Study. J Fam Violence. 2022 May 11;1–11.

24. Koerner SS, Shirai Y. The negative impact of global perceptions of and daily care-related family conflict on Hispanic caregivers: Familism as a potential moderator. Aging Ment Health. 2012;16(4):486–99.

25. Mausbach BT, Harmell AL, Moore RC, Chattillion EA. Influence of caregiver burden on the association between daily fluctuations in pleasant activities and mood: A daily diary analysis. Behav Res Ther. 2011;49(1):74–9.

26. Pickering CEZ, Yefimova M, Wang D, Maxwell CD, Jablonski R. Dynamic structural equation modelling evaluating the progressively lowered stress threshold as an explanation for behavioural symptoms of dementia. J Adv Nurs [Internet]. 2021 [cited 2022 Mar 29];n/a(n/a). Available from: https://onlinelibrary.wiley.com/doi/abs/10.1111/jan.15173

27. Koerner SS, Kenyon DYB. Understanding “good days” and “bad days”: Emotional and physical reactivity among caregivers for elder relatives. Fam Relat. 2007 Jan;56(1):1–11.

28. Bangerter LR, Liu Y, Kim K, Zarit SH. Adult day services and dementia caregivers’ daily affect: the role of distress response to behavioral and psychological symptoms of dementia. Aging Ment Health. 2021 Jan;25(1):46–52.

29. van Knippenberg RJ, de Vugt ME, Ponds RW, Verhey FRJ, Myin-Germeys I. Emotional reactivity to daily life stress in spousal caregivers of people with dementia: An experience sampling study. PLoS ONE. 2018;13(4).

30. Shirai Y, Koerner SS. Examining the influence of care-recipient resistance on family caregiver emotional and physical well-being: Average frequency versus daily fluctuation. J Appl Gerontol. 2018;37(2):203–27.

31. Thorpe J, Forchhammer BH, Maier AM. Adapting Mobile and Wearable Technology to Provide Support and Monitoring in Rehabilitation for Dementia: Feasibility Case Series. JMIR Form Res. 2019 Oct 17;3(4):e12346.

32. Ebner-Priemer UW, Welch SS, Grossman P, Reisch T, Linehan MM, Bohus M. Psychophysiological ambulatory assessment of affective dysregulation in borderline personality disorder. Psychiatry Res. 2007 Apr 15;150(3):265–75.

33. Colombo D, Fernández-Álvarez J, Suso-Ribera C, Cipresso P, Valev H, Leufkens T, et al. The need for change: Understanding emotion regulation antecedents and consequences using ecological momentary assessment. Emotion. 2020;20(1):30.

34. Jones A, Remmerswaal D, Verveer I, Robinson E, Franken IHA, Wen CKF, et al. Compliance with ecological momentary assessment protocols in substance users: a meta-analysis. Addict Abingdon Engl. 2019 Apr;114(4):609–19.

35. Soyster PD, Bosley HG, Reeves JW, Altman AD, Fisher AJ. Evidence for the Feasibility of Person-Specific Ecological Momentary Assessment Across Diverse Populations and Study Designs. J Pers-Oriented Res. 2019;5(2):53–64.

36. Sliwinski MJ. Measurement-burst designs for social health research. Soc Personal Psychol Compass. 2008;2(1):245–61.

37. Bai Z, Luo S, Zhang L, Wu S, Chi I. Acceptance and Commitment Therapy (ACT) to reduce depression: A systematic review and meta-analysis. J Affect Disord. 2020 Jan 1;260:728–37.

38. Smyth JM, Juth V, Ma J, Sliwinski M. A slice of life: Ecologically valid methods for research on social relationships and health across the life span. Soc Personal Psychol Compass. 2017;11(10):e12356.

39. Verbakel E, Tamlagsrønning S, Winstone L, Fjær EL, Eikemo TA. Informal care in Europe: findings from the European Social Survey (2014) special module on the social determinants of health. Eur J Public Health. 2017 01;27(suppl_1):90–5.

40. Mehl MR, Conner TS. Handbook of research methods for studying daily life. New York, NY, US: The Guilford Press; 2012. xxvii, 676 p. (Handbook of research methods for studying daily life).
